# Multiplex Matrix Metalloproteinases Analysis in the Cerebrospinal Fluid Reveals Potential Specific Patterns in Multiple Sclerosis Patients

**DOI:** 10.3389/fneur.2018.01080

**Published:** 2018-12-18

**Authors:** Massimiliano Castellazzi, Daniela Ligi, Elena Contaldi, Davide Quartana, Mattia Fonderico, Luca Borgatti, Tiziana Bellini, Alessandro Trentini, Enrico Granieri, Enrico Fainardi, Ferdinando Mannello, Maura Pugliatti

**Affiliations:** ^1^Department of Biomedical and Specialty Surgical Sciences, University of Ferrara, Ferrara, Italy; ^2^Interdepartmental Research Center for the Study of Multiple Sclerosis and Inflammatory and Degenerative Diseases of the Nervous System, University of Ferrara, Ferrara, Italy; ^3^Department of Biomolecular Sciences, University “Carlo Bo” of Urbino, Urbino, Italy; ^4^School of Medicine, University of Ferrara, Ferrara, Italy; ^5^Department of Neuroscience and Rehabilitation, Azienda Ospedaliero-Universitaria di Ferrara, Ferrara, Italy; ^6^University Center for Studies on Gender Medicine, University of Ferrara, Ferrara, Italy; ^7^Department of Experimental and Clinical Biomedical Sciences, University of Florence, Florence, Italy

**Keywords:** multiple sclerosis, cerebrospinal fluid, matrix metalloproteinases, sex, aging, blood-brain-barrier, neuroinflammation, neurodegeneration

## Abstract

**Background:** Matrix metalloproteinases (MMPs) are pleiotropic enzymes involved in extracellular protein degradation and turnover. MMPs are implicated in the pathogenesis of many neurological diseases, including multiple sclerosis (MS).

**Objective:** To search the level of MMPs in the cerebrospinal fluid (CSF) of MS patients and detect possible disease-specific patterns.

**Methods:** CSF samples from 32 MS patients and, from 15 control subjects with other inflammatory neurological diseases (OIND) were analyzed. The Bio-Plex Pro Human MMP 9-Plex Panel (Bio-Rad) was used for the quantification of MMP-1, MMP-2, MMP-3, MMP-7, MMP-8, MMP-9, MMP-10, MMP-12, and MMP-13.

**Results:** CSF MMP-1 and MMP-12 levels were significantly reduced in MS as compared with OIND. In MS patients' CSF: (i) MMP-1 levels were significantly higher in women vs. men; (ii) MMP-10 concentrations were higher in patients with CSF-restricted IgG oligoclonal bands, and (iii) MMP-7 levels were increased in patients with longer disease duration. In the OIND group MMP-7 and MMP-12 levels significantly and directly correlated with age.

**Conclusions:** Our study contributes to investigating the role of MMPs in MS, with regard to CSF immunological features and disease duration. Sex-specific differences were also detected in MMPs CSF levels.

## Introduction

In multiple sclerosis (MS) the migration of immunocompetent cells into the central nervous system (CNS) requires the opening of the blood-brain barrier (BBB) (1), a mechanism in which proteins degradation represents a crucial step (2). Matrix metalloproteinases (MMPs) are pleiotropic calcium-requiring and zinc-containing proteolytic enzymes involved in extracellular matrix (ECM) degradation and turnover (3). MMPs are members of the metzincins family, so named for the presence of a conserved Methionine residue at the active site and for the use of a zinc ion in the enzymatic reaction (4). Twenty-three enzymes, encoded by 24 genes, have been identified in humans and included in this family (5). MMPs comprise both secreted and membrane-associated enzymes, and are classified into five subgroups according to their structure: collagenases (MMP-1, MMP-8, and MMP-13), stromelysins (MMP-3 and MMP-10), gelatinases (MMP-2 and MMP-9), a more heterogeneous subgroup containing matrilysin (MMP-7), enamelysin (MMP-20), macrophage metalloelastase (MMP-12), and MMP-19, and membrane-type MMP (MT-MMP) (MT-MMP-1-4 and stromelysin-3, MMP-11) (6). In the functional development of the CNS MMPs play a fundamental role in neurogenesis, axonal guidance, synaptic plasticity, learning and memory (7), and have been reported in association with ischemia/hypoxia, epilepsy, and seizure, Huntington's disease, vascular dementia, Parkinson's disease, malignant gliomas (8). Abnormal concentrations of MMPs have been reported in MS and its animal models, and in different phases of disease course and progression (7).

A distinctive pattern of MMPs expression in different cellular populations was reported. MMP-11, MMP-26 and MMP-27 were enriched in B cells, while MMP-15, MMP-16, MMP-24, and MMP-28 were prominent in T lymphocytes. Of interest was the enrichment of a majority of MMPs members in monocytes: MMP-1, MMP-3, MMP-9, MMP-10, MMP-14, MMP-19, and MMP-25 (9). Moreover, MMP-12 deficiency ameliorated a virus-induced murine model of MS (10). Likely due to the larger reagent availability, gelatinases (MMP-2 and MMP-9) represent the most studied MMPs in MS. In particular, MMP-9 levels were higher in relapsing remitting as compared to progressive MS (11) and active MMP-2 and MMP-9 were reciprocally associated to disease remission and activity, respectively (12, 13). In addition, gelatinases were described altered in response to both first and second line disease modifying therapies (interferon-beta 1a and natalizumab, respectively) in MS patients (14, 15).

We aimed to search the level of nine MMPs in the cerebrospinal fluid (CSF) of MS patients comparing it with a population of controls and with multiplex assay, in the attempt to ultimately investigate the role of MMPs in the disease.

## Methods

### Study Design

This study retrospectively included 32 patients with relapsing-remitting MS according to the currently accepted criteria (16). At the time of sample collection (a) disease duration was defined as the time (months) elapsed between clinical onset and study time; (b) the presence of CSF-restricted IgG oligoclonal bands (OCB) was recorded as intrathecal immunoglobulin synthesis; (c) the number of T2-weighted lesions on magnetic resonance imaging (MRI) scans was recorded. Patients affected by other inflammatory neurological diseases (OIND) were selected as controls. None of the study subjects underwent treatment with immunosuppressants or immune-modulating drugs, including steroids, at the time of sampling.

In MS group, lumbar puncture was performed as part of the diagnostic work-up for a suspect of MS, subsequently all patient were ‘naïve' for disease modifying treatment.

The study was approved by the Committee for Medical Ethics in Research of Ferrara and written consent to study participation was obtained from all subjects.

### Sample Handling

CSF samples were obtained from MS and OIND patients through diagnostic lumbar puncture. Cell-free CSF was obtained after centrifugation at 3,000 rpm at 20°C for 15 min. Supernatants were collected, under sterile conditions, in aliquots of 500 μl, coded, frozen and stored at −80°C until assay. As routine CSF examination, intrathecal immunoglobulin synthesis was investigated through isoelectric focusing followed by IgG-specific immunoblotting in paired CSF and serum samples (17).

### Magnetic Resonance Imaging Analysis

All MS patients underwent brain MRI scans on a 1.5-Tesla MRI unit (GE Signa Horizon, General Electric Medical Systems, Milwaukee, WI, USA) within 48 h after CSF sampling. Routinely used T1-weighted axial spin echo images were obtained ~10 min after intravenous injection of 0.1 mmol/kg of Gadopentetic acid (Gd-DTPA) in each patient. Lesions showing contrast-enhancement on T1-weighted scans were defined as active (Gd+). All neuroimaging data were independently reviewed by two neuroradiologists (L.B. and E.F., with 15 and 20-years experience, respectively) blinded to the patients' clinical and laboratory data. Discrepancies between readers were resolved by consensus adjudication.

### MMP Multiplex Assay

MMPs concentrations in CSF were determined in all samples through the Pro™ HumanMMP 9-plex Assay (including: MMP-1, MMP-2, MMP-3, MMP-7, MMP-8, MMP-9, MMP-10, MMP-12, and MMP-13), a multiplex suspension immunomagnetic assays based on the use of fluorescently dyed magnetic beads covalently conjugated with monoclonal antibodies specific for the target proteins (Bioplex, Bio\\Rad Labs, Hercules, CA, USA). As indicated by the manufacturer, the lower detection limit was 1.0 pg/mL, while the mean intra-assay variability was 10%. Levels of all analytes were determined using a Bio-Plex 200 array reader, based on Luminex X-Map Technology (Bio\\Rad Labs, Hercules,CA, USA). The protein concentrations (expressed as pg/mL) were calculated through a standard curve.

### Statistical Analysis

Frequencies (percent) and chi-square statistics were reported for categorical variables and mean [standard deviation (SD)] and t-student statistics for continuous variables as descriptives. The log-normal transformation was applied to non-normally distributed data. Mann–Whitney *U*-test was used to compare MS vs. OIND patients and MS subgroups each other. Spearman's rank test was used for correlation. Analysis of covariance (ANCOVA) with MMPs CSF levels as dependent variables, status (MS vs. OIND) as fixed factor, and sex and age at study time as covariates. Two-tail *p*-values were reported and statistical significance was set at *p* < 0.05. The Statistical Package for the Social Sciences (SPSS®) version 19 for Windows and OSX (SPSS Inc., IBM®, Somers, New York, USA) and Prism® (GraphPad Software Inc.) were used for statistical analysis.

## Results

The study was conducted on 47 individuals, 32 patients with a diagnosis of MS, and 15 control subjects suffering from OIND. The main clinical-demographic features of the study population are reported in Table 1. The women:men ratio was marginally higher in the MS than in the OIND group (3:1 vs. 0.9:1; *p* = 0.056), and mean age at study time was significantly higher in the OIND than in the MS group (58.6 vs. 39.4 years, *p* = 0.000004).

**Table 1:** Clinical and demographic main features of study population.

	**MS (*n* = 32)**	**OIND (*n* = 15)**	***p***
Sex, female: *N* (%)	24 (75)	7 (46.7)	0.056^*a*^
Age, years: mean (SD)	39.4 (11.6)	54.9 (14.5)	0.0002^*b*^
Age, years: median (min-max)	42.0 (19–60)	57.0 (28–78)	–
Disease duration, months: mean (SD)	34.6 (67.2)	–	–
Number of MRI T2 lesions: mean (SD)	12.9 (10.0)	–	–
MRI Gd enhancement, yes: N (%)	16 (50.0)	–	–
CSF-restricted IgG OCB, positive: N (%)	24 (75.0)	–	–

a *Chi-square test*.

b *U-test*.

*CSF, cerebrospinal fluid; Gd, gadolium; MRI, magnetic resonance imaging; MS, multiple sclerosis; OCB, oligoclonal bands; OIND, other inflammatory neurological diseases; SD, standard deviation*.

*OIND: acute inflammatory demyelinating polyneuropathy (4 patients), MRI-negative clinically isolated syndromes (3), chronic inflammatory demyelinating polyneuropathy (2), other inflammatory disease of CNS (1), vascular ischemic disorder (1), isolated myelitis (1), polineuropahy (1), isolated optic neuritis (1), systemic lupus erythematosus (1)*.

### MMP CSF Levels in MS Patients and Controls

The percentage of samples with detectable levels of each MMPs was similar in MS and OIND (Table 2). MMP-3 resulted undetectable in almost all samples and MMP-13 was present only in a small percentage of both MS patients and controls. MMP-3 and MMP-13 were therefore excluded from further analysis.

**Table 2:** Percentage of cerebrospinal fluid samples with detectable levels of MMPs.

	**MS; n(%)**	**OIND; n(%)**
MMP-1	31 (96.9)	15 (100)
MMP-2	32 (100)	15 (100)
MMP-3	1 (3.1)	0 (0)
MMP-7	32 (100)	15 (100)
MMP-8	30 (93.8)	14 (93.3)
MMP-9	20 (62.5)	5 (33.3)
MMP-10	31 (96.9)	15 (100)
MMP-12	31 (96.9)	15 (100)
MMP-13	11 (34.4)	3 (20)

*MMPs, matrix metalloproteinases; MS, multiple sclerosis; OIND, other inflammatory neurological diseases*.

CSF MMP-1 and MMP-12 levels were significantly higher in OIND than in MS patients after adjusting for sex and age at study time (ANCOVA; *p* = 0.009 and *p* = 0.012, respectively) (Table 3). No further statistically significant differences were found between CSF MMPs concentrations comparing MS and OIND subjects.

**Table 3:** Adjusted mean (SD) (95%CI) concentrations (pg/ml) of MMPs in MS vs. OIND patients.

	**Adjusted mean (SD)^*a*^**		
	**MS**	**OIND**	***p***
MMP-1	70.8 (5.1)	67.0 (8.0)	0.009
	(60.6, 81.0)	(51.0, 83.0)	
MMP-2	195.4 (20.4)	160.3 (32.1)	0.441
	(154.2, 236.6)	(95.6, 225.0)	
MMP-7^b^	3.89 (0.15)	3.20 (0.24)	0.052
	(3.58, 4.20)	(2.71, 3.69)	
MMP-8^b^	1.41 (0.14)	1.83 (0.21)	0.132
	(1.14, 1.69)	(1.39, 2.26)	
MMP-9^b^	1.63 (0.27)	2.47 (0.65)	0.447
	(1.06, 2.19)	(1.13, 3.81)	
MMP-10^b^	2.28 (0.08)	2.72 (0.13)	0.512
	(2.11, 2.45)	(2.47, 2.98)	
MMP-12^b^	1.21 (0.13)	1.52 (0.20)	0.012
	(0.95, 1.47)	(1.11, 1.92)	

*MMPs, matrix metalloproteinases; MS, multiple sclerosis; OIND, other inflammatory neurological diseases*.

a *Analysis of covariance (ANCOVA) marginal means: MMPs as dependent variable, status (MS vs. OND) as fixed factor, and sex and age at study time as covariates*.

b *After log-normal transformation of MMP concentration*.

### MMP CSF Levels and Sex in MS and Controls

**Figure 1:**
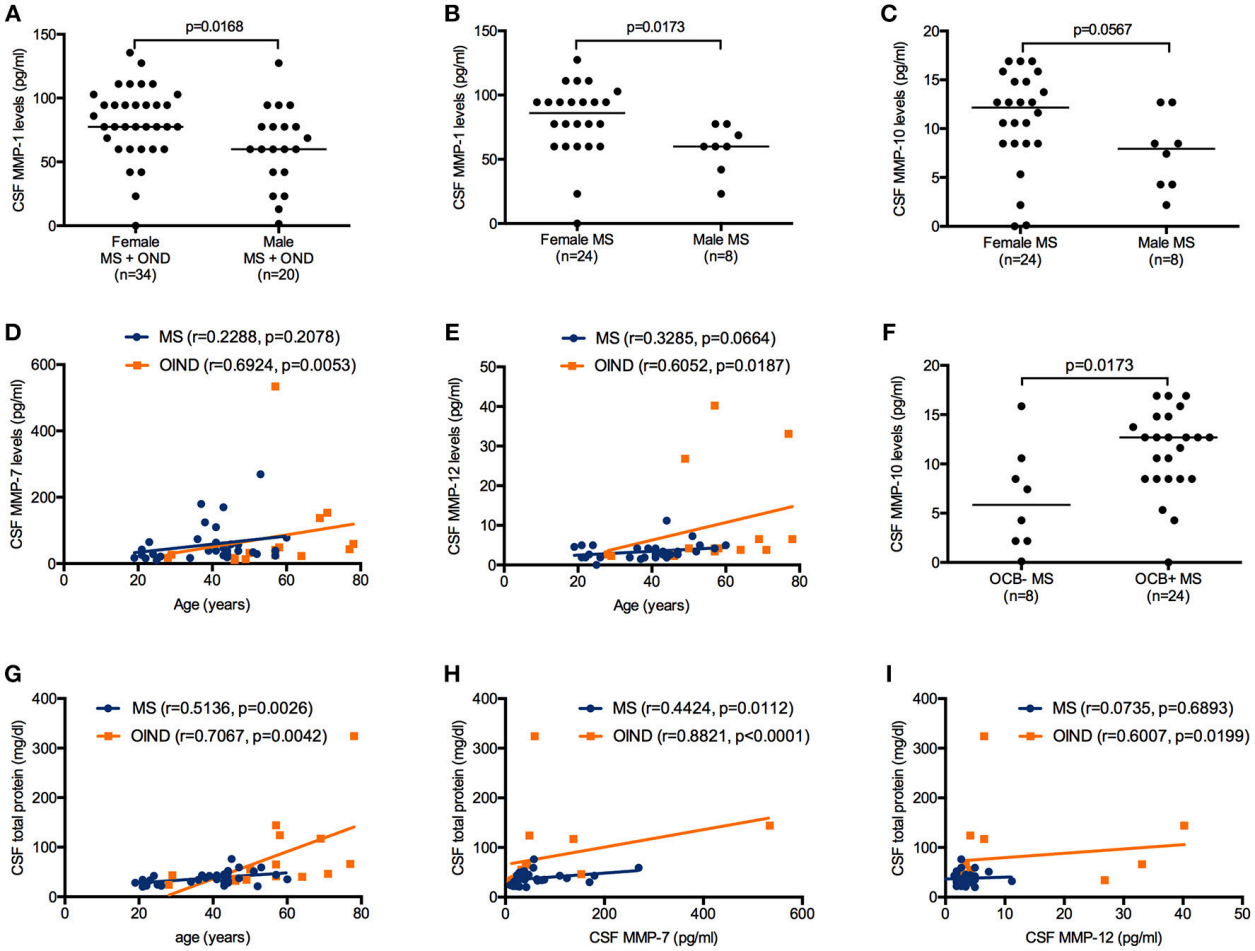
Matrix metalloproteinases (MMPs) cerebrospinal fluid **(**CSF) levels in patients affected by multiple sclerosis (MS) and by other inflammatory neurological diseases (OIND) considered as a whole and classified according to clinical diagnosis and clinical features. MMP-1 levels were higher in female than in male patients considered as a whole (Mann–Whitney; *p* = 0.0168, **(A)**. MMP-1 levels were increased in female than in male MS patients (Mann–Whitney; *p* = 0.0173) **(B)**. MMP-10 levels were more elevated in female than in male MS patients, however the *p* value did not reach the significance (Mann–Whitney; *p* = 0.0567) **(C)**. MMP-7 and MMP-12 concentrations were positively correlated to age in the overall sample (Spearman; *r* = 0.2920 and *p* = 0.0321, *r* = 0.5674 and *p* < 0.0001, respectively) and in the OIND group (Spearman; *r* = 0.6924 and *p* = 0.0053, *r* = 0.6052 and *p* = 0.0187, respectively) **(D,E)**. MMP-10 levels were higher in MS patients positive for the presence of IgG oligoclonal bands (OCB+) than in negative (OCB-) (Mann–Whitney; *p* = 0.0173) **(F)**. CSF total protein levels were positively correlated to age in MS and OIND (Spearman; *p* = 0.0026 and *p* = 0.0042, respectively) **(G)**. In CSF: MMP-7 and total protein concentrations were positively correlated in MS and OIND (Spearman; *p* = 0.0112 and *p* < 0.0001, respectively) **(H)**, while MMP-12 positively correlated to total protein in OIND (Spearman; *p* = 0.0199) **(I)**. Each point represents a single observation. Horizontal bars indicate medians **(A–C,F)**.

MMP-1 CSF levels were significantly increased in female than in male patients (MS + OIND) (Mann–Whitney; *p* = 0.0070, Figure 1A). There was no significant difference in age between female and male MS patients at the time of sample withdrawal (40.4 vs. 36.6 years, respectively; *p* = 0.5277). CSF MMP-1 levels were higher in female than in male MS patients (Mann-Whitney; *p* = 0.0173) (Figure 1B). Moreover, an almost reached statistical significance was found for CSF MMP-10 higher concentrations in female than in male MS subjects (Mann–Whitney; *p* = 0.0567) (Figure 1C). No further statistically significant difference was detected for other MMP CSF concentrations by sex nor by status (data not shown).

### MMP CSF Levels and Age in MS and Controls

CSF MMP-12 concentrations were positively correlated to age in the overall sample (Spearman; *r* = 0.5155 and *p* = 0.0002) while CSF MMP-7 levels and age correlation almost reached statistical significance (Spearman; *r* = 0.2788 and *p* < 0.0577). CSF MMP-7 and MMP-12 levels showed a positive correlation to patients' age (years) at study time only in the OIND group (Spearman; *r* = 0.6924 and *p* = 0.0053, *r* = 0.6052 and *p* = 0.0187, respectively) (Figures 1D,E). No other statistically significant correlations were found between MMPs levels and age in MS and OIND groups (data not shown).

### MMP CSF Levels and Intrathecal IgG Synthesis in MS Patients

Twenty-four (75%) MS patients were positive for CSF-restricted IgG OCB (OCB+ MS). OCB+ MS and OCB negative MS patients (OCB- MS) showed no differences in age at the time of sample withdrawal (38.6 vs. 42.0 years; *p* = 0.4597). MMP-10 CSF levels were higher in OCB+ than in OCB- MS patients (Mann–Whitney; *p* = 0.0173) (Figure 1F), while no other statistically significant differences were detected in other MMPs CSF concentrations between the two MS subgroups (data not shown).

### MMP and Total Protein CSF Levels in MS and Controls

CSF total protein levels were positively correlated to age in the overall samples (Spearman; *r* = 0.6310 and *p* < 0.0001) and in MS and OIND subgroups (Spearman; *r* = 0.5136 and *p* = 0.0026, *r* = 0.7067 and *p* = 0.0042, respectively) (Figure 1G). MMP-7 and total protein CSF concentrations were positively correlated in the overall samples (Spearman; *r* = 0.5303 and *p* = 0.0001) and in MS and OIND patients (Spearman; *r* = 0.4424 and *p* = 0.0112, *r* = 0.8821 and *p* < 0.0001, respectively) (Figure 1H). CSF MMP-12 levels positively correlated to CSF total protein concentrations in the overall samples (Spearman; *r* = 0.31353 and *p* = 0.0319) and in OIND subjects (Spearman; *r* = 0.6007 and *p* = 0.0199) (Figure 1I). No further statistically significant correlations were between MMPs levels and total CSF protein concentrations (data not shown).

### MMP CSF Levels and Disease Duration in MS Patients

MS patients with long disease duration (defined as more than 12 months) were older than patients with short disease duration (defined as 0–12 months) (48.5 vs. 38.6 years; *p* = 0.0009). The analysis of covariance (ANCOVA) was used to compare CSF MMPs levels in MS patients by dichotomised disease duration and adjusting for age at study time (Table 4). CSF MMP-7 concentrations only were found to be statistically greater in MS patients with long disease duration as compared to short disease duration (*p* = 0.034).

**Table 4:** Adjusted mean (95%CI) levels of MMPs (pg/ml) and disease duration (months) among 32 MS patients^a^.

	**MMP-1**	**MMP-2**	**MMP-7^**b**^**	**MMP-8^**b**^**	**MMP-9^**b**^**	**MMP-10^**b**^**	**MMP-12^**b**^**
**Disease duration (months)**							
0–12	74.2 (60.9, 87.6)	208.3 (157.1, 259.5)	3.5 (3.1, 3.8)	1.6 (1.3, 1.8)	1.6 (1.0, 2.2)	2.4 (2.2, 2.6)	1.2 (1.0, 1.4)
>12	76.7 (57.4, 96.0)	180.1 (106.2, 253.9)	4.3 (3.8, 4.7)	1.5 (1.1, 1.8)	1.9 (0.7, 3.1)	2.3 (2.0, 2.5)	1.0 (0.7, 1.3)
*p*	0.647	0.744	0.034	0.295	0.136	0.095	0.075

*MMPs, matrix metalloproteinases; MS, multiple sclerosis*.

a *ANCOVA marginal means (95%CI) with MMPs as dependent variable, disease duration (12 vs. > 12 months) as fixed factor and age at study time as covariate*.

b *After log-normal transformation of MMP concentration*.

### MMP CSF Levels and Magnetic Resonance Features in MS

Detectable CSF levels of any MMP under study did not differ in MS patients with MRI evidence of disease activity vs. non active MRI (data not shown). CSF MMPs levels were similar in Gd+ and in Gd- MS patients and were not associated with the number of T2-weighted lesions (data not shown).

## Discussion

To the best of our knowledge, for the first time, we report a reduced expression of MMP-1 and MMP-12 in the CSF of patients with MS as compared to subjects with OIND, after adjusting for sex and age at study time. A regulated MMP activity contributes to physiological synaptic plasticity, while dysregulated activity can produce tissue injury. Allen and coll. demonstrated that the application of exogenous MMP-1, *in vitro*, stimulated dendritic arborization, while overexpression of MMP-1, *in vivo*, increased dendritic complexity and induced biochemical and behavioral endpoints (18). These data suggest an important role of MMP-1 in maintaining neuronal plasticity. Conflicting results were previously reported on the role of MMP-12 within the CNS. In fact, MMP-12 were associated to both demyelination (19) and remyelination (20). Despite of the detrimental role of almost all MMPs, some of them, including MMP-1, have been associated to physiological and neurogenesis processes comprising myelination and proliferation and differentiation of neural progenitor cells (21, 22). In light of this, the reduced expression of some MMPs in the CNS of MS patients could reflect a minor capability in tissue repairing.

In our study MS population, MMP-7 CSF levels were found to be directly associated with disease duration, even after adjusting for age at study time. MMP-7 was found in association with a protective and/or anti-inflammatory role in MS, and clearly in the early phases of the disease as compared to neurodegeneration. It was previously shown that MMP-7 was lower in children with MS as compared to controls and that the treatment with interferon-β was able to increase serum MMP-7 concentrations (23).

We found that MMP-10 CSF levels were higher in OCB+ than in OCB- MS patients. Detection of CSF IgG OCB remains both a fundamental support to the diagnosis and a prognostic factor in MS (16). The significance of OCB in the pathogenesis and/or prognosis of MS is still unclear, however MS patients with no IgG OCB in their CSF are more likely to show better prognosis (24). The production of IgG OCB seems to be sustained by the presence of CNS infiltrating plasma cells in MS lesions and to the presence of tertiary lymphoid structures in the meningeal compartment (25). These structures recapitulate lymphoid follicle-like features providing an excellent microenvironment for the interaction of B and T cells. MMP-10 was described as mainly expressed by monocytes and by stimulated T-cells, however, a lower expression was also demonstrated in both stimulated and unstimulated B-cells (9). In this perspective, in OCB+ MS patients, CSF elevated MMP-10 concentrations could reflect a remarkable presence of resident T and B cells as compared to OCB- MS subjects.

Surprisingly, in the present study we did not find any correlation with MRI markers of disease activity and progression. This is in contrast with our previous evidences that the active forms of MMP-2 and MMP-9 were correlated to MRI evidence of disease remission or activity, respectively (12, 13). An explanation of this apparently conflicting result might be ascribed to different techniques used in the first two studies, in which only the active enzyme forms and not the total amount of both MMP-2 and MMP-9 were assessed. Of note, many MMPs are expressed as inactive zymogens which require extracellular activation (26).

Gender-specific patterns are an interesting research focus in MS due to the high and increasing women:men ratio (27). Women generally have an earlier onset of disease, counterbalanced by a less progression of disability than men (28). In the overall study population, and in MS patients, CSF levels of MMP-1 were higher among women than men. An effect of gender on MMPs was reported in previous studies. MMP-9 serum levels were higher in female than in male population (29) and MMP-2 mRNA was reported to be reduced in response to the female hormone 17β-estradiol (30). Moreover, 17β-estradiol decreased degradation of tight junction proteins and suppressed the up-regulation of MMPs expression leading a protective role of estrogenin in BBB disruption using an *in vitro* and *in vivo* model (31). Our result is also in line with a previous study in which MMP-3 levels were associated to male sex and to a low level of MMP-1 (32).

In the overall sample we found an association between aging and CSF MMP-12 levels, while in the OIND group both MMP-7 and MMP-12 were positively associated to age. Previous study showed that MMP-7 was associated with a reduction in N-methyl-D-aspartate receptor stimulated calcium flux and authors speculated on a protective role of this MMP in age-related neurodegenerative diseases (33). Moreover, in the overall samples MMP-7 and MMP-12 were positively correlated to CSF total protein concentrations indicating a relationship between the levels of MMP-7 and the increased BBB permeability that occur in aging (17). Increased MMP-12 were previously reported in mice brain during aging, suggesting that the enzyme could enhance the age-associated neuroinflammation (34). When patients were grouped according to the diagnosis, the correlations between MMPs and age were confirmed only in the control group suggesting that the relationship between high levels of some MMPs and patient's age becomes more significant with aging.

The main limitations of this study were certainly the small sample size of the OIND group and the lack of a control group including non-inflammatory neurological diseases. However, the differences detected for MMP-1 and MMP-12 suggest patterns specifically associated to MS.

Taken together our results paint a novel and interesting picture of the role of MMPs in MS, with regard to other immunological features of CSF (e.g., OCB and BBB permeability), but also disease duration. Sex-specific differences were also detected in MMPs CSF levels.

Future studies in larger MS populations and on serum samples are however needed to explain (i) the detailed function and interrelationship of MMP-1, MMP-7, MMP-10, and MMP-12 in MS and (ii) the potential use of these molecules as biological markers during neuroinflammation -degeneration and in tissue repairing (35).

## Data Availability Statement

Datasets are available on request: The raw data supporting the conclusions of this manuscript will be made available by the authors, without undue reservation, to any qualified researcher.

## Author Contributions

MC participated in the design and coordination of the study, analysis and interpretation of data, and drafted the manuscript. DL participated in the acquisition and analysis of samples and revised the manuscript. EC, DQ, and MF participated in the acquisition and analysis of clinical data and revised the manuscript. LB participated in the acquisition and analysis of MRI data and revised the manuscript. TB, AT, and EG were involved in the analysis and interpretation of data and manuscript revision. EF, FM, and MP participated in the design of the study, in the analysis and interpretation of data, and drafted the manuscript. All authors read and approved the final manuscript.

### Conflict of Interest Statement

The authors declare that the research was conducted in the absence of any commercial or financial relationships that could be construed as a potential conflict of interest.
